# D-Dimer-Driven Anticoagulation Reduces Mortality in Intubated COVID-19 Patients: A Cohort Study With a Propensity-Matched Analysis

**DOI:** 10.3389/fmed.2021.631335

**Published:** 2021-02-04

**Authors:** Apostolos K. Tassiopoulos, Sima Mofakham, Jerry A. Rubano, Nicos Labropoulos, Mohsen Bannazadeh, Panagiotis Drakos, Panagiotis Volteas, Nathaniel A. Cleri, Leor N. Alkadaa, Anthony A. Asencio, Anthony Oganov, Wei Hou, Daniel N. Rutigliano, Adam J. Singer, James Vosswinkel, Mark Talamini, Charles B. Mikell, Kenneth Kaushansky

**Affiliations:** ^1^Department of Surgery, Renaissance School of Medicine, Stony Brook, NY, United States; ^2^Division of Vascular Surgery, Department of Surgery, Renaissance School of Medicine, Stony Brook, NY, United States; ^3^Department of Neurosurgery, Renaissance School of Medicine, Stony Brook, NY, United States; ^4^Department of Family, Population and Preventive Medicine, Renaissance School of Medicine, Stony Brook, NY, United States; ^5^Department of Emergency Medicine, Renaissance School of Medicine, Stony Brook, NY, United States; ^6^Department of Medicine, Renaissance School of Medicine, Stony Brook, NY, United States

**Keywords:** D-dimer-driven anticoagulation, anticoagulation, d-dimer, thrombotic complications, hypercoagulability, COVID-19

## Abstract

**Objective:** Examine the possible beneficial effects of early, D-dimer driven anticoagulation in preventing thrombotic complications and improving the overall outcomes of COVID-19 intubated patients.

**Methods:** To address COVID-19 hypercoagulability, we developed a clinical protocol to escalate anticoagulation based on serum D-dimer levels. We retrospectively reviewed all our first 240 intubated patients with COVID-19. Of the 240, 195 were stratified into patients treated based on this protocol (ON-protocol, *n* = 91) and the control group, patients who received standard thromboprophylaxis (OFF-protocol, *n* = 104). All patients were admitted to the Stony Brook University Hospital intensive care units (ICUs) between February 7th, 2020 and May 17, 2020 and were otherwise treated in the same manner for all aspects of COVID-19 disease.

**Results:** We found that the overall mortality was significantly lower ON-protocol compared to OFF-protocol (27.47 vs. 58.66%, *P* < 0.001). Average maximum D-dimer levels were significantly lower in the ON-protocol group (7,553 vs. 12,343 ng/mL), as was serum creatinine (2.2 vs. 2.8 mg/dL). Patients with poorly controlled D-dimer levels had higher rates of kidney dysfunction and mortality. Transfusion requirements and serious bleeding events were similar between groups. To address any possible between-group differences, we performed a propensity-matched analysis of 124 of the subjects (62 matched pairs, ON-protocol and OFF-protocol), which showed similar findings (31 vs. 57% overall mortality in the ON-protocol and OFF-protocol group, respectively).

**Conclusions:** D-dimer-driven anticoagulation appears to be safe in patients with COVID-19 infection and is associated with improved survival.

**What This Paper Adds:** It has been shown that hypercoagulability in patients with severe COVID-19 infection leads to thromboembolic complications and organ dysfunction. Anticoagulation has been variably administered to these patients, but it is unknown whether routine or escalated thromboprophylaxis provides a survival benefit. Our data shows that escalated D-dimer driven anticoagulation is associated with improved organ function and overall survival in intubated COVID-19 ICU patients at our institution. Importantly, we found that timely escalation of this anticoagulation is critical in preventing organ dysfunction and mortality in patients with severe COVID-19 infection.

## Introduction

Thrombosis is a major cause of morbidity and mortality in severe COVID-19 illness. Deep vein thrombosis (DVT) (1), pulmonary embolism (PE) (2, 3), cerebral infarction (4), and myocardial infarction (MI) (5, 6) have all been reported in patients severely ill because of COVID-19 (7–9). Additionally, vascular access including dialysis catheters (10) and extracorporeal membrane oxygenation (ECMO) circuits (11) also fail at higher rates. D-dimer levels, a marker of fibrin breakdown (12) and intravascular clot burden (13), are increased in many patients with COVID-19. The degree of elevation correlates with disease severity (14) and it is particularly high in patients who died (15). Furthermore, widespread microthrombosis in the kidneys (16) and lungs (17), and other organs has been reported at autopsy. These observations strongly indicate that coagulopathy in COVID-19 infection contributes to organ dysfunction and mortality.

Anticoagulation appears to improve outcomes in critically ill COVID-19 patients, according to an early report from China (18), as well as a larger series from New York (19). However, two reports from France reported unexpected PE despite therapeutic anticoagulation (20, 21), presumably due to the severity of the hypercoagulability. Recently, a group in Italy reported that non-critically ill patients did not benefit from elevated doses of anticoagulation, though details were lacking about what agents and doses were administered (22). Additionally, an Italian group reported improved mortality in a retrospective cohort that received an “intermediate” dose of low molecular weight heparin (LMWH, 40–60 mg twice daily) (23). These findings were echoed in a report by five centers in New York City in which both prophylactic and therapeutic anticoagulation were associated with improved mortality, although there was not a significant difference between these groups (*p* = 0.07) (24). Despite these promising signs, a large randomized trial of therapeutic anticoagulation (NIH ACTIV-4) recently paused enrollment for lack of efficacy in critically-ill patients (25). At present, no randomized data is available to support one approach over another.

Despite the dearth of data, several professional societies have promulgated guidelines on anticoagulation in COVID-19. The American Society of Hematology (ASH) currently recommends against the use of therapeutic doses of heparin or LMWH in these patients in the absence of confirmed or suspected VTE (26). However, the International Society of Thrombosis and Hemostasis (ISTH) also recommends routine thromboprophylaxis with subcutaneous unfractionated heparin (UFH) or LMWH, with consideration for intermediate doses of LMWH in high-risk patients. The guidelines also state to consider a 50% increase in the dose of thromboprophylaxis in obese patients (27). Finally, current NIH treatment guidelines also indicate that there is not enough data available to recommend the use of anticoagulants at higher doses in these patients (28). Thus, new data are urgently needed.

During the early phase of the pandemic, our group observed severe arterial and venous thrombosis in COVID-19 patients despite routine, standard low-dose thromboprophylaxis. Based on these early observations, we hypothesized that escalated thromboprophylaxis for hypercoagulability (as indicated by elevated D-dimer levels) would limit thrombotic complications and improve outcomes in COVID-19 infection. We also suspected that many patients with increasing D-dimer levels had occult thromboses, either PE, or clots in other vascular beds. Therefore, we developed a protocol to escalate the level of anticoagulation, based on serum D-dimer levels, measured on a daily basis.

In the current report, we reviewed all of our COVID-positive, intubated patients (n = 240) admitted between February 7th, 2020 and May 17, 2020. We describe the 91 intubated COVID-19 intensive care unit (ICU) patients who received thromboprophylaxis based on this protocol, during the first wave of the pandemic in New York. At the peak of the pandemic, two of five ICUs at our institution had agreed on the escalated anticoagulation protocol; the 91 reported patients were admitted to these ICUs, based on pure chance availability of beds. This random assignment (a sort of “experiment of nature”) gives us the opportunity to understand whether escalated anticoagulation had a benefit, vs. routine care. We compared these patients to a cohort of 104 ICU patients who either received routine thromboprophylaxis or started full dose anticoagulation when standard clinical indications (e.g. DVT, PE) became apparent. Both groups were admitted to the hospital and ICU during the same time period, and their clinical care was otherwise similar.

## Methods

### Ethics Statement

This study was a retrospective chart review of a COVID-19 patient database. Stony Brook University Committee on Research in Human Subjects approved the study protocol and supervised all study procedures, in accordance with state and federal regulations, with a waiver of informed consent.

### Target Population and Data Sources

**Figure 1:**
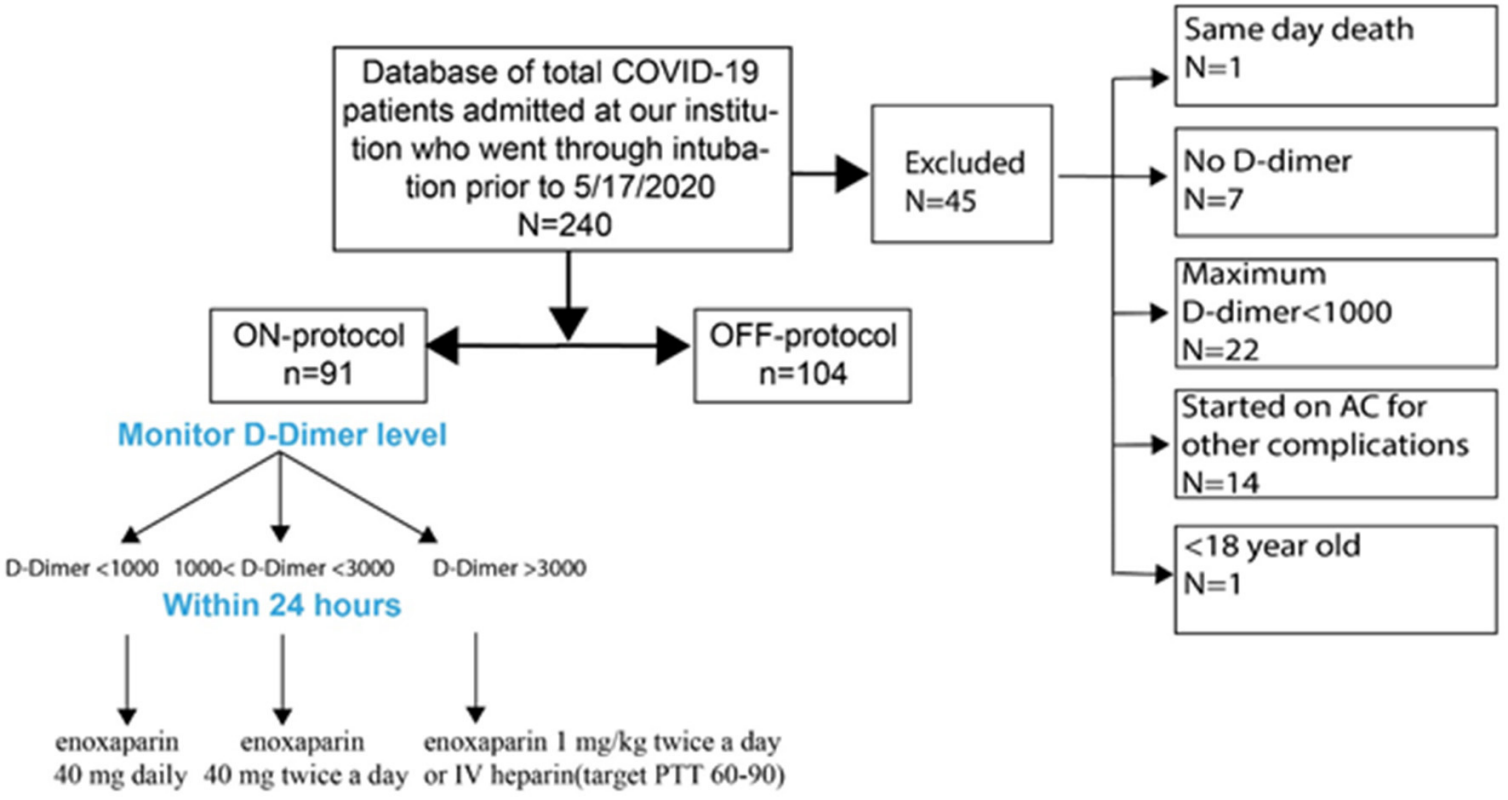
Patient selection algorithm and the anticoagulation protocol.

We identified all intubated COVID-19 patients admitted to Stony Brook University Hospital between February 7, 2020 and May 17, 2020 with a positive RT-PCR test for SARS-CoV-2 (Figure 1). Our initial screen identified 240 intubated patients. We then applied our inclusion/exclusion criteria:

### Inclusion Criteria

1. Age ≥ 18
2. COVID-19 infection with a positive RT-PCR test
3. Respiratory failure requiring endotracheal intubation
4. At least two D-dimer measurements after intubation
5. D-dimer elevation to >1,000 ng/mL during ICU course.

### Exclusion Criteria

1. Oral anticoagulation prior to and on admission
2. Therapeutic anticoagulation initiated because of cardiac arrhythmia
3. Pregnancy or delivery within 2 weeks of intubation
4. Death in the Emergency Department
5. Known bleeding diathesis or hypercoagulability
6. D-dimer levels <1,000 ng/mL throughout hospitalization
7. No D-dimer levels sent during hospitalization.

Patients were then stratified to ON- and OFF-protocol. ON-protocol patients met the following criteria:

1. Anticoagulation administration was based on D-dimer level, in the following sliding scale:
  a. D-dimer <1,000 ng/mL: enoxaparin 40 mg daily
  b. D-dimer ≥ 1,000 ng/mL but <3000 ng/mL: enoxaparin 40 mg twice a day
  c. D-dimer ≥ 3,000 ng/mL: enoxaparin 1 mg/kg twice a day, or therapeutic anticoagulation with IV heparin (target PTT 60-90), based on physician preference.
2. Escalation of anticoagulation occurred within 24 h of a change in the D-dimer level.

OFF-protocol patients met the inclusion criteria but did not meet ON-protocol requirements. In general, physicians adhered to the protocol, with some bias toward administration of anticoagulation at lower D-dimer levels than prescribed (Supplementary Figure 1). In many cases, enoxaparin was used despite creatinine elevation; there was no obvious increase in hemorrhagic complications (see Table 1). In many OFF-protocol patients, anticoagulation was administered eventually, for other clinical reasons or more than 24 h after changes in D-dimer. No patients in this group were initially administered anticoagulation at ICU admission, and we excluded patients given heparin or other anticoagulation because of cardiac arrhythmia from this study. Additionally, patients who did not meet inclusion criteria are excluded from this report. Adjudication of patients to the two groups was done by three authors and classification was by consensus.

**Table 1:** Cause of death.

	**Cause of death**					
**Groups**	**MOF**	**Suspected PE**	**Hypoxic respiratory failure**	**MI**	**CNS complications**	**Aspiration**
ON-protocol	14	4	5	1	1	0
OFF-protocol	42	13	2	1	2	1
TOTAL	56	17	7	2	3	1

### Random Assignment to ICUs

The chance circumstance that made the comparison between ON- and OFF-protocol groups possible was adoption of escalated anticoagulation in two of five ICUs at our institution. In the remaining three ICUs, anticoagulation was administered in routine fashion (see Results). Assignment to different ICUs was a random process, based on bed availability.

### Chart Review

We reviewed each chart and collected the following data:

1. Demographics
2. Dates of admission and intubation
3. Comorbidities
4. Laboratory data
5. Adverse events from COVID-19 (death, thromboembolic phenomena, renal failure). We documented “suspected PE” in patients who were previously on stable ventilatory settings and suddenly developed acute respiratory deterioration with increased needs for ventilatory support and concomitant circulatory collapse, not able to be attributed to other causes (sepsis, myocardial infarction, pneumothorax, mucous plug, etc.). Due to the extreme precautions to control the dissemination of the SARS-CoV-2 virus along with patients' hemodynamic instability, we decided to treat those patients preemptively as having PE, without any confirmatory imaging test.
6. SOFA score—this score was calculated with lab values sent at the time of intubation and for 24 h subsequently (29). If a lab value was not available immediately, it was carried forward from admission labs. Phenylephrine was converted to norepinephrine equivalents as suggested by Lambden (30).
7. Clinically significant bleeding defined as:
  1. Gastrointestinal bleeding requiring transfusion of at least two units of red blood cells (RBCs);
  2. Hemoglobin <7 mg/dL and transfusion of at least two units of RBCs;
  3. Intracranial bleeding or
  4. Other major bleeding requires transfusion including massive hemoptysis, hematuria, retroperitoneal hematoma, intraperitoneal, or intrathoracic bleeding.
8. Long-term outcomes (death, discharge from hospital) are reported if available. For all 195 patients, 4 months of follow-up data were available. To this date, 96.4% (188/195) of patients have either been discharged from the hospital or deceased. All patients were included in the Kaplan-Meier analysis.

### Data Analysis

#### Laboratory Analysis

We generated time series data in MATLAB representing D-dimer levels and other laboratory values, time-locked to three main dates: admission date, intubation date, and anticoagulation starting date. We collected laboratory values for all patients (except for one patient with missing laboratory data) and calculated the mean and standard error (SE) for both groups. The ACL TOP Hemosil D-dimer HS (high sensitivity) test was used to assess D-dimer levels.

#### Statistics

Statistical analyses were performed using SPSS 21.0 software (SPSS Inc., Chicago, Ill) and in-house developed coding in MATLAB. The significance level for all tests was 0.05. All reported *P* values were calculated two-sided. The primary endpoint was death. Secondary endpoints included discharge.

Data were reported as group means, along with the two-tailed Student's T-statistic for several labs (D-dimer, BUN, creatinine). We had hypothesized that these specific values would be different and thus no multiple comparisons correction is appropriate. Non-parametric analysis was performed to compare the means of maximum D-dimer, creatinine, BUN, and SOFA score. Other categorical variables such as hypertension, chronic kidney disease, chronic obstructive pulmonary disease (COPD), sex, and diabetes were compared using the χ2 test. Two-sample *T*-test or Mann-Whitney *U*-tests were used for continuous variables as indicated based on normal distribution vs. skewness of factors.

Survival and its association with measured factors were evaluated using Kaplan-Meier models. A log-rank test was used to compare survival between groups. There was no missing data regarding survival measures. We used Cox proportional-hazards regression models to estimate the predictors of survival. The multivariable Cox regression model included participation in the protocol, gender, age, SOFA score, and BMI. Entry-level for multivariable analysis was *P* < 0.1. The multivariable model had an excellent fit with *P* < 0.001. Hazard ratios were calculated to estimate independent predictors of survival.

#### Propensity-Score Matched Analysis

We performed a propensity score-matched analysis of 122 of the subjects to isolate the effect of anticoagulation on outcome. We used logistic regression to calculate a propensity score (31) and matched cases using the “Greedy algorithm” (32). Regression model variables included age, gender, BMI, SOFA score, heart disease, diabetes, and hypertension, and excluded pairs with the distance of PS score > 0.01. Additional variables were excluded because of relatively low numbers.

## Results

### Study Population

After initial screening, from a total of 240 patients admitted to Stony Brook University Hospital ICUs between February 7, 2020, and May 17, 2020, 195 patients were included for analysis. The exclusion criteria can be found in Figure 1. Patients were randomly assigned to one of five ICUs on admission, based on bed availability. During the first wave of COVID-19 cases at our institution, there was a wide inter-practitioner variation in thromboprophylaxis. However, physicians in two of our five ICUs rapidly agreed on a protocol for escalated anticoagulation, based on D-dimer levels, because of the clinical observation of severe thromboembolic events (see Methods). Hospital leadership promulgated official guidelines for the care of COVID-19 patients on March 25th, leading to the relative uniformity of care; nearly all patients included in the study were intubated after this date (**Figure 3A**). We stratified the 195 patients into ON-protocol (*n* = 91) and OFF-protocol (*n* = 104) groups. Note that both groups were admitted to the hospital and ICU during the same time period. The mean ages in each group were similar (57.7 vs. 61.7, *P* = 0.079), as were other demographic features, and antiviral drugs and steroids were used at similar rates (Table 2, Supplementary Table 1). Most patients were given hydroxychloroquine and steroids; rates were similar in ON-protocol and OFF-protocol groups. However, very few patients received remdesivir (13%, 18.6% ON-protocol, and 8.6% OFF-protocol). However, remdesivir did not change the mortality in this subgroup (Supplementary Figure 2, left panel). Exclusion of patients who were treated with remdesivir did not change the study overall results (Supplementary Figure 2, right panel). We calculated initial SOFA scores for each patient upon intubation. The distribution of SOFA scores is shown in Supplementary Figure 3 (ON protocol: 6.19; OFF protocol, 6.97, Mann-Whitney *U*-test, *P* = 0.21). We also accounted for this difference with a propensity-matched analysis (see below).

**Table 2:** Patient demographics and adverse events.

	**OFF-Protocol (*N* = 104)**	**ON-Protocol (*N* = 91)**	***P* value**
Age—year (mean ± SE)	61.7 ± 1.6	57.7 ± 1.6	0.079
Male (%)	76 (73.07)	65 (71.14)	0.79
SOFA score (mean ± SE)	6.97 ± 0.24	6.19 ± 0.21	0.21
Max D-dimer (mean ± SE)	12343 ± 1318	7553 ± 972	0.005
Admission Creatinine (mean ± SE)	1.41 ± 0.15	1.114 ± 0.08	0.1
BMI (mean ± SE)	30.04 ± 0.63	30.27 ± 0.6	0.79
Intubation (%)	104 (100)	91 (100)	
Death (%)	61 (58.6)	25 (27.47)	<0.001
Discharged (%)	39 (37.5)	62 (68.13)	<0.001
Days from intubation to death	17 ± 1.63	19 ± 2.52	
PE(including suspected PE) /DVT (%)	24 (23.07)	9 (9.8)	<0.014
Received Transfusion (%)	49 (47.11)	45 (49.4)	0.74
COMORBIDITIES			
HTN (%)	58 (55.7)	46 (50.5)	0.46
COPD (%)	5 (4.8)	6 (6.5)	0.58
Heart failure	6 (5.7)	2 (2.1)	0.2
Diabetes	29 (27.88)	30 (32.9)	0.44
CKD (%)	7 (6.7)	3 (3.2)	0.27

*Group characteristics and adverse events. Groups were similar at baseline. The ON-protocol group had significantly lower D-dimer levels, fewer deaths, and fewer pulmonary emboli and deep vein thromboses. Categorical values were compared with chi-square statistics. Independent-samples Mann-Whitney U tests were used for noncategorical variables which could not be assumed to be distributed normally*.

### ON-Protocol Patients had Low Mortality

**Figure 2:**
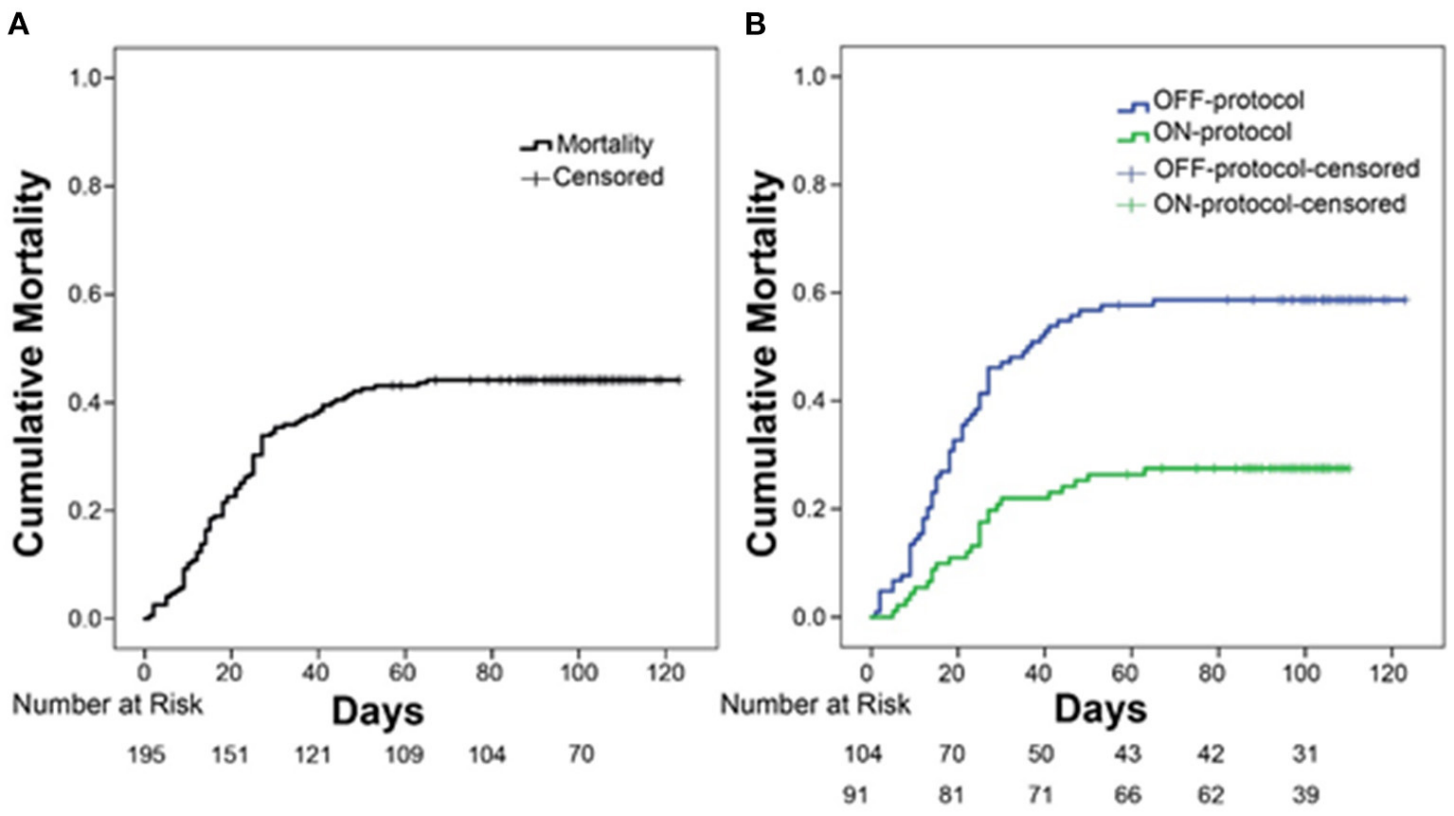
Protocol-driven anticoagulation is associated with significantly lower mortality. **(A)** Overall mortality in intubated patients with COVID-19 infection who were admitted to the ICU. **(B)** Comparison of overall mortality between ON-protocol group (green line, *N* = 91) and OFF-protocol group (blue line, *N* = 104) (log-rank test, *P* < 0.001).

Overall cumulative mortality (with a minimum of 4 months of follow up for all the patients) for ICU patients with severe COVID-19 was 44% (86/195, Figure 2A, Table 2). Kaplan-Meier survival analysis demonstrated that ON-protocol group patients had significantly lower mortality rates compared to the OFF-protocol group (Figure 2B, overall mortality 27.47 vs. 58.6%, *P* < 0.001; Table 2).

**Figure 3:**
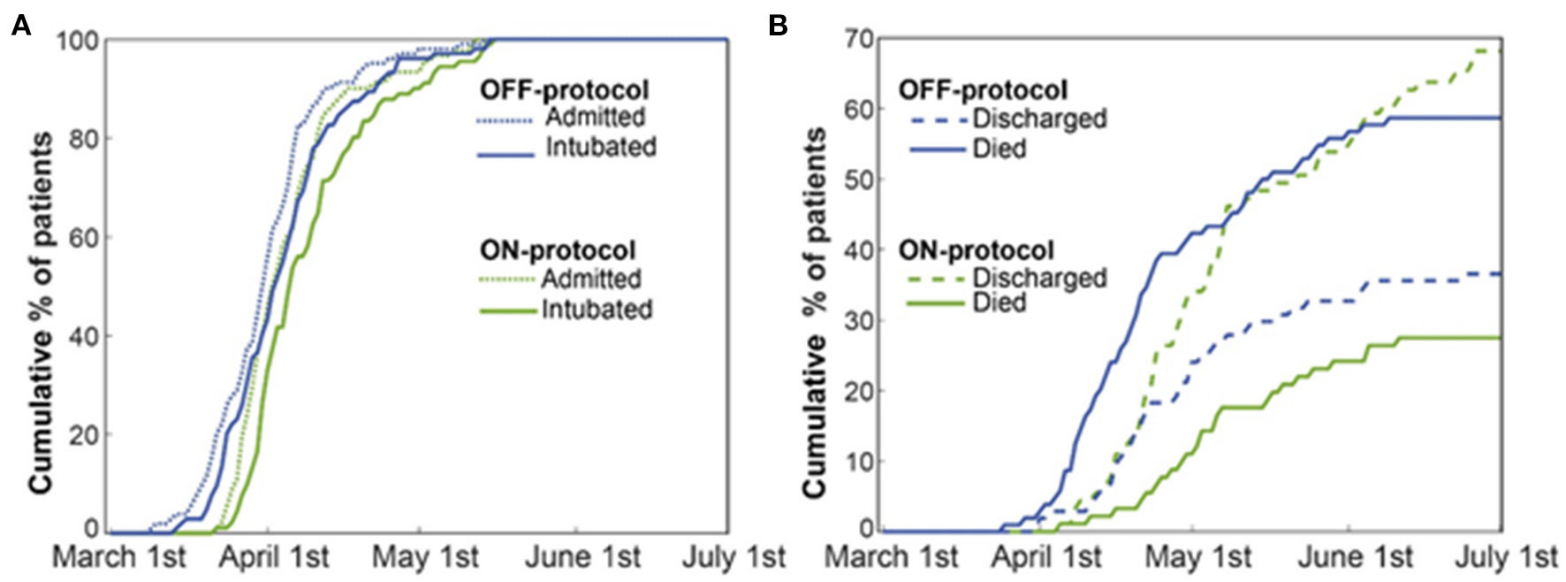
The ON-protocol and OFF-protocol groups were admitted at similar time periods but with drastically different outcomes in terms of mortality and discharged rates. **(A)** The admission and intubation timeline of both ON (green) and OFF-protocol (blue) groups are shown. **(B)** For both the ON- (green lines) and OFF-protocol (blue lines) groups, we plotted the accumulated percentages of the discharged and expired patients. Dashed and solid lines, respectively, represent the accumulated percentages of discharged and expired patients in each group. The overall mortality rate is 58.65% in the OFF-protocol group compared to the 27.47% in the ON-protocol group, while patients in the ON-protocol group were discharged at a much higher rate (69.23% compared to 37.5% in the OFF-protocol group).

In univariate survival analysis, patients in the OFF-protocol group (*P* < 0.0001), male patients (*P* = 0.051) with age greater/equal to 70 years (*P* < 0.001), and SOFA score greater/equal to seven (*P* < 0.001) were each associated with lower rates of survival. The multivariable analysis shows that OFF-protocol group membership was an independent predictor of higher mortality (hazard ratio [HR], 2.33; 95% confidence interval [CI], 1.4-3.75; *P* = 0.0001). In the multivariable analysis of mortality, male sex (HR, 1.79; 95% CI, 1.04-3.07; *P* = 0.034), SOFA score greater/equal to seven (HR, 2.16; 95% CI, 1.33-3.5; *P* = 0.002), and age over 70 (HR, 2.02; 95% CI, 1.28-3.17; *P* = 0.002) were also predictors of poor outcome. Importantly, both groups were admitted to the hospital and ICU during the same time period (Figure 3A). Cumulative mortality increased rapidly in the OFF-protocol group, while discharges were more common in the ON-protocol group (Figure 3B).

### Propensity-Matched Analysis

**Figure 4:**
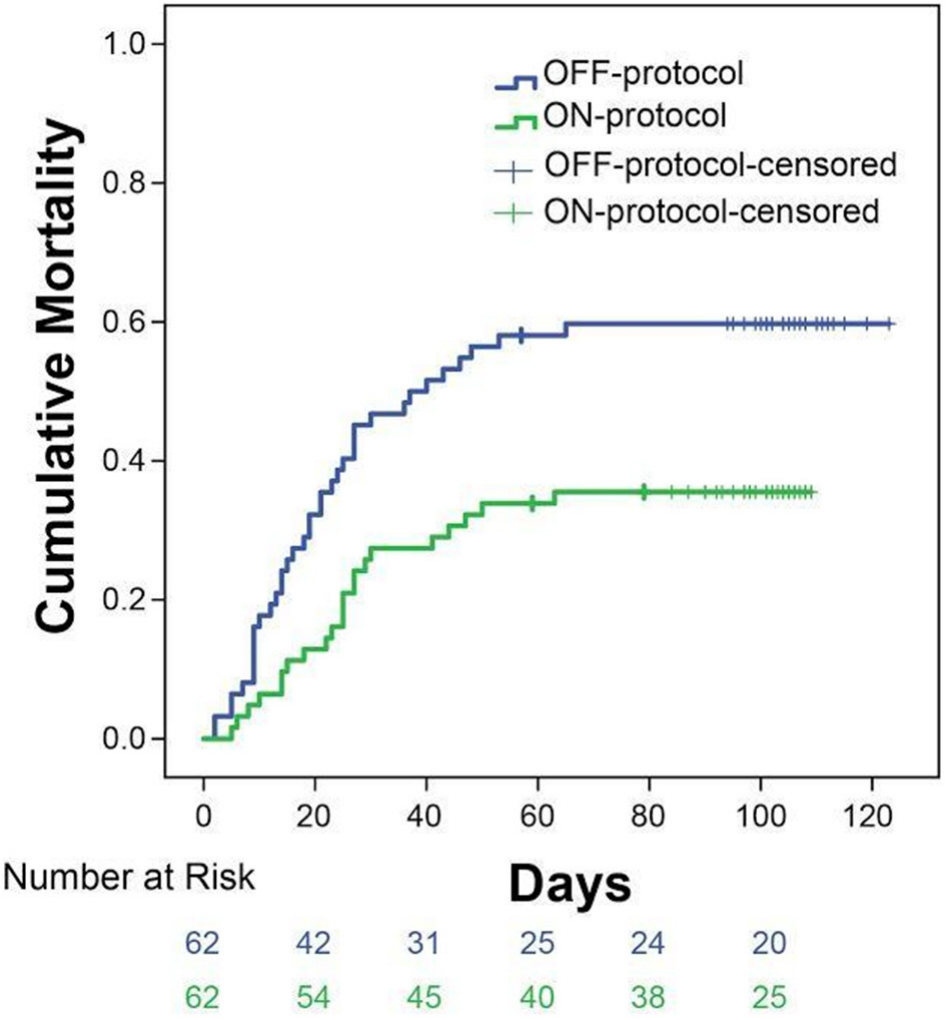
Subgroup analysis of 124 patients matched from ON-protocol (*n* = 62) and OFF-protocol groups (*n* = 62) exhibits similar results as the whole group analysis. Sixty two pairs of ON and OFF-protocol patients were analyzed on age, gender, BMI, SOFA score, heart disease, diabetes, and hypertension with the distance of a PS score ≤ 0.01. The other comorbidity variables were not used because the values were ≤ 10. The mortality rates in these groups are 31% (ON-protocol) vs. 57% (OFF-protocol, *P* = 0.0061).

To account for possible differences between the study groups, we performed a propensity score-matched analysis. We were able to match 124 patients within a propensity score of < 0.01. Patients who received ON-protocol anticoagulation had a mortality of 31 vs. 57% mortality in the OFF-protocol matched cohort (Figure 4). Importantly, Kaplan-Meier curves of ON-protocol and OFF-protocol groups for the propensity-matched groups were similar to the Kaplan-Meier curve obtained from the whole sample.

### Anticoagulation Per-Protocol Robustly Controls D-Dimer Levels and Kidney Function

**Figure 5:**
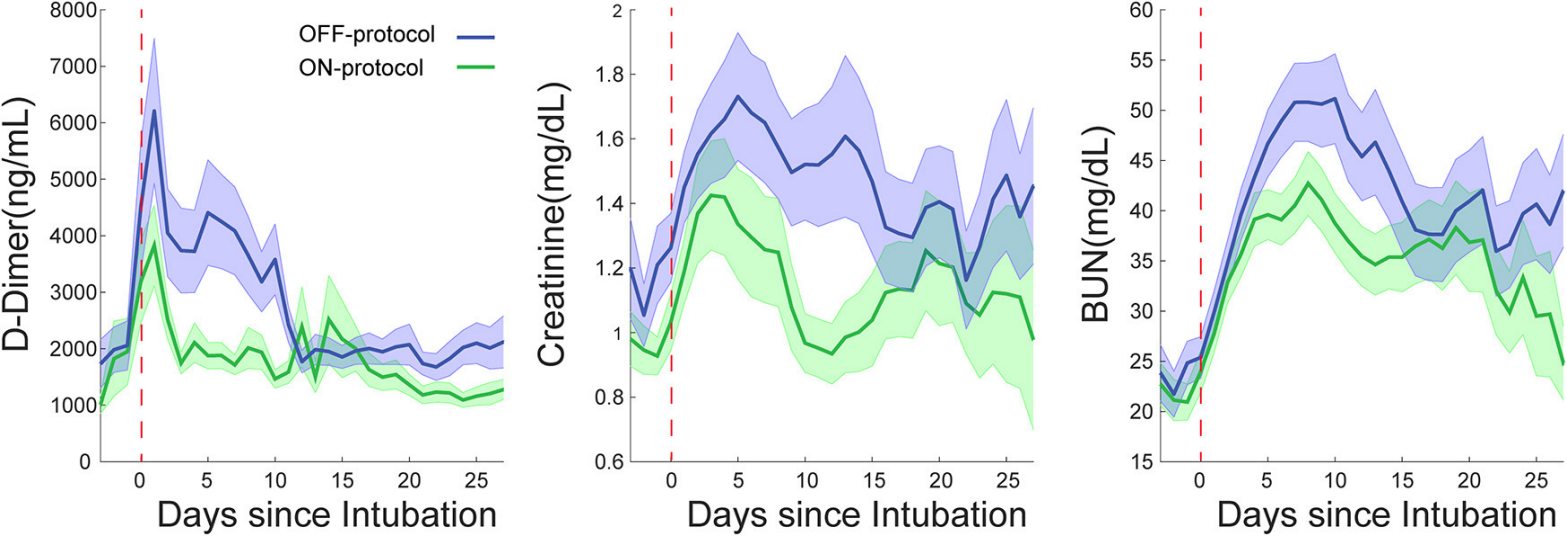
Escalated, D-dimer driven anticoagulation (ON-protocol group) is associated with improved critical laboratory values in multiorgan dysfunctions in COVID-19 intubated patients. The early start of AC in the ON-protocol group was associated with significant changes in the course of the disease in intubated patients. The green and blue lines represent the mean of D-dimer, creatinine and BUN for ON- and OFF-protocol groups over thirty days. Notice the elevated level of these laboratory values in the first two weeks following intubation, which is associated with many of mortalities in the OFF-protocol group. The shaded area represents the SE of the mean. These analyses are time-locked to the intubation date marked by the red dashed line.

In addition to lower mortality, the ON-protocol group displayed a higher hospital discharge rate compared to the OFF-protocol group (Figure 3B). To uncover the potential mechanism underlying this fast recovery and improved outcome, we analyzed serial D-dimer levels in patients who were ON- and OFF-protocol, as well as creatinine and BUN values over time. Figure 5 shows how these laboratory values (mean ± SE), time-locked to the intubation date, develop over days after intubation. These results revealed that early anticoagulation robustly controlled D-dimer levels. During the first two weeks after intubation, D-dimer, creatinine, and BUN were especially elevated in OFF-protocol patients. This is a critical time period associated with most of the deaths in COVID-19 intubated patients, and most of the mortality in the OFF-protocol patients occurred during this time period (associated with the steep slope of the mortality curve in Figure 3B). The mean maximum D-dimer level was 7,553 ng/mL (median 4028 ng/mL) for ON-protocol patients, and 12,343 ng/mL (median 7030 ng/mL) for OFF-protocol (Mann-Whitney *U*-test, *P* = 0.001, Figure 5, left panel). Patients who were ON-protocol also had lower creatinine levels (mean of maximum value for ON-protocol = 2.2 mg/dL, median = 1.23 mg/dL, SE = 0.22, and OFF-protocol = 2.81 mg/dL, median = 1.98 mg/dL, SE = 0.24, Mann-Whitney *U*-test, *P* < 0.019, Figure 5, middle panel). In contrast, BUN (ON-protocol = 66.23 mg/dL [median = 47 mg/dL], SE = 4.68, OFF-protocol = 77.63 mg/dL (median = 68.3 mg/dL), SE = 5.07, *P* < 0.126) did not achieve statistically different values in the aggregate, although the trend was different (Figure 5). Anticoagulation, therefore, was associated with superior kidney function and overall outcome.

### Adverse Events

The ON-protocol group experienced a lower incidence of thromboembolic complications. Four patients who expired had suspected PE. Two patients were diagnosed with segmental PEs on imaging, and three patients were found to have DVTs. One patient was diagnosed with cerebral infarction after extubation. Six patients had arterial thromboses: four patients who were diagnosed with non-ST elevation myocardial infarction (NSTEMI), one with splenic infarcts and one with lower extremity arterial embolism.

By contrast, in the OFF-protocol group, 13 patients who expired had suspected PE. Five surviving patients had imaging-confirmed PE and six were diagnosed with DVT (total of 23% PE/DVT compared to 9.8% in ON-protocol patients, *p* = 0.014). Eleven patients had arterial thromboses: two patients suffered from ischemic stroke, seven patients had clinically significant MI, one acute limb ischemia and one mesenteric ischemia.

### Bleeding Complications Were Similar Between Groups

Bleeding complications were frequent but similar between groups. Nine patients in the ON-protocol group developed upper or lower GI bleeding, manifesting as melena, blood in the orogastric tube or hematochezia, and four more required transfusions due to bloody respiratory secretions, hemothorax, mediastinal, and tracheostomy site bleeding. A total of 19 patients experienced a hemoglobin drop to <7 mg/dL at some point during hospitalization and one patient had a hemorrhagic stroke.

In the OFF-protocol group, nine patients developed GI bleeding presenting in the same way as the ON-protocol patients, and nine required transfusions for retroperitoneal bleeding, hematuria, hemothorax, and bloody respiratory secretions. A total of 21 patients developed a hemoglobin <7 mg/dL and two experienced intracranial bleeding.

PRBC transfusion unit requirements were very similar between two groups (ON-protocol, median 0, range 0-18, mean 2.38, SE 0.39; OFF-protocol, median 0, range 0–24, mean 2.9, SE 0.48; Mann Whitney *U*-test, *P* = 0.989).

## Discussion

Our study results indicate that an early-onset escalating thromboprophylaxis protocol based on daily D-dimer level is associated with significantly fewer thrombotic complications, preserved kidney function, and improved mortality in intubated patients with severe COVID-19 infection. The mortality in the ON-protocol cohort was 27%, compared to 58% in the OFF-protocol group, although this comparison needs to be made with caution, given the fact that this study is not a randomized trial, and it is possible there were unobserved differences between the groups that account for the differences in mortality. Nonetheless, the propensity-matched analysis also supports our core hypothesis, which should be confirmed in larger, randomized trials. The outcomes in the ON-protocol group are superior to those described in other published reports (33) and is probably due to both prevention of large-vessel thrombosis and improvement of kidney function, possibly by prevention of microthrombosis. Many fewer patient deaths in the ON-protocol group were attributed to large arterial or venous thrombotic complications, and clinically significant hemorrhage was not different between groups. On the contrary, thirteen patients in the OFF-protocol group died during their ICU course from probable PE, despite being administered standard low-dose thromboprophylaxis.

Our findings are generally consistent with the reported high incidence of thrombotic complications in COVID-19 ICU patients (7–9). At present, there is no official guidance about anticoagulation in COVID-19, except for previously promulgated guidelines (26–28, 34). There are reports indicating some benefit of anticoagulation particularly in critically ill patients with COVID-19 infection (18, 19), but the anticoagulation type is variable, and timing of onset is not reported, so comparisons are difficult. Of note, Paranjpe et al. (19) reported a similar mortality benefit for anticoagulation in intubated patients (29 vs. 62% mortality), though no further details about these subgroups are available in their manuscript. The report of Nadkarni and colleagues which did not identify a significant difference between therapeutic and prophylactic anticoagulation made no effort to propensity match or establish that the groups receiving prophylactic and/or therapeutic anticoagulation were similar. Thus, their data should not be over-interpreted to claim there is no benefit to therapeutic anticoagulation. By contrast, our data, while not conclusive, support the view that escalated anticoagulation may be appropriate when the D-dimer level rises. We designed our protocol to escalate the intensity of anticoagulation based on D-dimer levels because of the reported association of higher D-dimer levels to increased mortality (15, 35). We believed that early thromboprophylaxis would control the prothrombotic effect of severe COVID-19 infection, prevent early death from thrombotic complications, and limit the extent of microthrombi, thus preventing patient progression to multi-system organ failure (MSOF). This notion is supported by our analysis which indicates that ON-protocol anticoagulation controls the D-dimer level, prevents the occurrence of thromboembolic complications, preserves organ perfusion (as measured by preserved renal function), and is the only independent predictor of patient survival, and was accomplished without an increased risk of the need for transfusion. Our data underscore the importance of the timing of the anticoagulation. This early D-dimer driven escalation could also explain why the ON-protocol mortality we observed is lower than what has been reported in the literature for intubated ICU patients, whether they received anticoagulation or not (15, 33).

## Limitations

This study has several limitations, inherent to the single-institution, retrospective design with a small sample size and the fact that it is subject to residual confounding. Since the two groups of patients were treated in different ICUs, we cannot eliminate with certainty the possibility that other aspects of patient's care might explain the difference in the outcome. When these patients were becoming critically ill, our institution was in the rapidly escalating pandemic curve. In this phase bed availability and the patient assignment were a random event. COVID-19 treatment protocols have been otherwise consistent in our hospital for critically ill patients throughout the pandemic. The anticoagulation protocol was addressed and implemented institution-wide later allowing for this difference in care. However, we did not observe any major differences in the management other than the protocol for anticoagulation, and the propensity-matched analysis was similar. Nevertheless, propensity-matched analysis has its limitations, including the fact that in developing the propensity scores, important variables that could have affected the outcome may have been inadvertently omitted. Moreover, only two-thirds of the patients were able to be matched. Thus, while the use of anticoagulation was associated with improved outcomes, causality cannot be proved. Furthermore, creatinine was slightly more elevated in the OFF-protocol group, although the mean SOFA scores at protocol initiation were not significantly different. We do think comparisons between the two groups should be made with caution, but the outcomes of the OFF-protocol group are similar to those described in the literature (15, 19, 33). Additionally, our study did not include any comparison with patients not receiving anticoagulation, and we did not do further analysis to compare the two types of anticoagulation regimens that were used; low-molecular-weight heparin and unfractionated heparin. Our cohort was relatively overweight (mean BMI of 30); it is possible that a thinner cohort would have fewer thromboembolic complications. However, obesity is now a well-established risk factor for severe COVID-19 disease (36–38), and at least one report describes the mean BMI in their cohort as 29 (39). Thus our cohort's BMI is probably fairly representative of other critically-ill patients. Finally, although the differences in outcomes in the groups studied herein are impressive, the fact that pulmonary embolism/thrombosis symptoms frequently overlap those of severe COVID-19 infection, and that imaging was underutilized to prevent unnecessary staff exposure, might have led to underdiagnosis of thromboembolic complications. This fact might explain the higher D-dimer levels and higher mortality rate that was observed in the OFF-protocol group. And while recent commentaries call for controlled trials of anticoagulation in patients with COVID-19 (40), we believe the dramatic difference in outcomes revealed by these data should be carefully considered in designing and awaiting results of a double-blinded, controlled trial. These findings and the success of this protocol that has the longest follow-up among all published studies, provide a window toward understanding the mechanisms driving excessive thrombosis and its treatment in this disease.

## Conclusion

Protocol-driven anticoagulation was safe and effective in the treatment of a cohort of COVID-19 patients and associated with significantly lower mortality and improved kidney function. Our findings should be validated in a larger randomized, controlled trial.

## Data Availability Statement

The data supporting the conclusions of this article will be made available by the authors upon reasonable request.

## Ethics Statement

The studies involving human participants were reviewed and approved by Stony Brook University Committee on Research in Human Subjects. Written informed consent for participation was not required for this study in accordance with the national legislation and the institutional requirements.

## Author Contributions

AT, SM, CM, NL, MB, DR, JR, PD, PV, JV, MT, and KK: study conception and design. AT, JR, MB, DR, MT, and JV: designed the clinical protocol. SM, WH, and MB: data analysis and making figures. AT, SM, CM, NL, MB, DR, JR, PD, PV, NC, LA, AS, AO, WH, JV, MT, and KK: planning the data analysis and data interpretation. PD, PV, NC, LA, AS, AO, SM, JR, CM, and AA: data acquisition. AT, CM, SM, NL, MT, and KK: drafting the manuscript. All authors: critically revising the manuscripts. All the authors gave the final approval of the version to be published.

## Conflict of Interest

The authors declare that the research was conducted in the absence of any commercial or financial relationships that could be construed as a potential conflict of interest.
