# Supplementary material for: D-Dimer-Driven Anticoagulation Reduces Mortality in Intubated COVID-19 Patients: A Cohort Study With a Propensity-Matched Analysis
Source: Front Med (Lausanne). 2021 Feb 4;8:631335. doi: 10.3389/fmed.2021.631335 (PMC7902033; doi:10.3389/fmed.2021.631335)
Supplement: Supplementary file 1 [file Image_1.pdf]

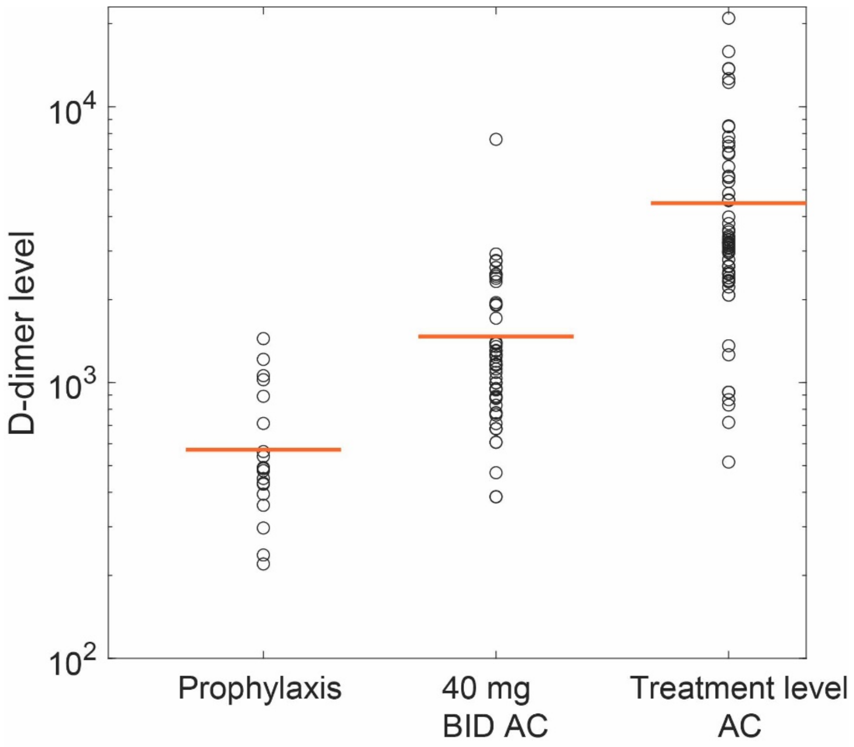

Supplementary Figure 1. Measured D-dimer level of ON-protocol patients corresponding to the start of the protocol. The average D-dimer level for ON-protocol groups that were administered with prophylaxis was 570.88 (SE=68.7), those who were administered with 40mg BID 1492 (SE=167) and the treatment level 4728(SE=515).

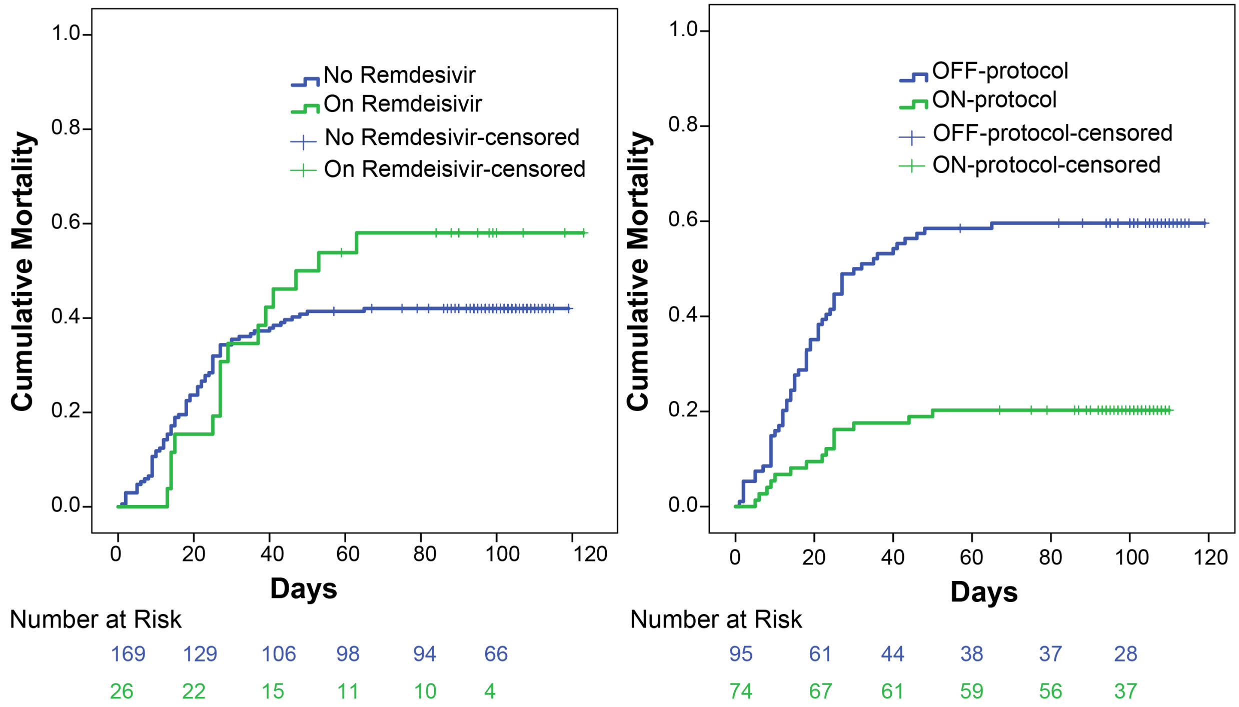

Supplementary Figure 2. Administration of remdesivir in our patient population did not affect mortality. A) In the studied patient population 26/195 (13.3%) received remdesivir, including nine patients (8.6%) OFF-protocol and 17 patients (18.6%) ON-protocol. 57% of patients who got remdesivir died, as opposed to 44% overall mortality of all patients. There was no statistically significant difference in the mortality of patients who received remdesivir, (log rank, *P*=0.362). **B)** Excluding the subgroup of patients (26/195) who received remdesivir did not affect the overall result of our study. The ON-protocol group still had substantially lower mortality than the OFF-protocol group (overall mortality 20.27% versus 59.57%, *P*<0.0001).

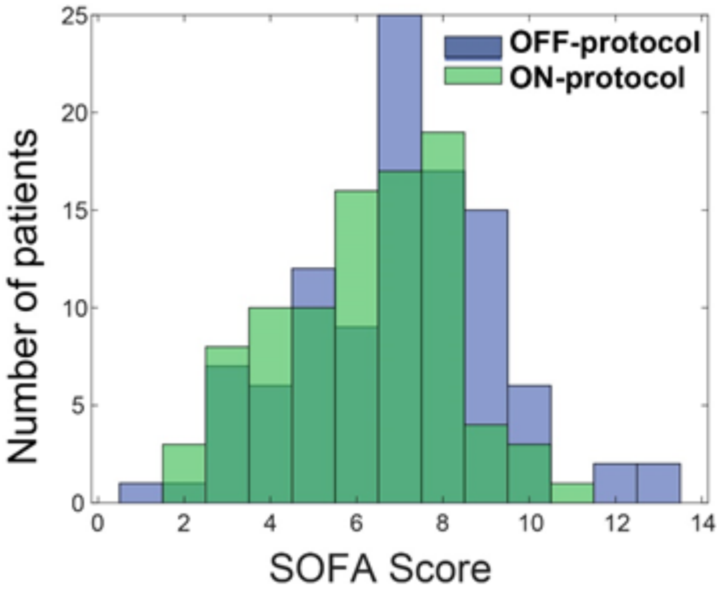

Supplementary Figure 3. SOFA Scores for ON-protocol and OFF-protocol groups are in similar ranges.

Supplementary Table 1. The Medication list for ON- protocol and OFF-protocol groups.

Medication	ON-protocol	OFF-protocol	*P* value
Hydroxychloroquine	80(87.91)	94(90.38)	0.57
All steroids	77(84.61)	96(92.3)	0.09
Dexamethasone	3(3.29)	8(7.6)	0.18
Hydrocortisone	17(18.68)	27(25.96)	0.22
Methylprednisolone	77(84.61)	85(81.73)	0.59
Prednisone	38(41.75)	33(31.73)	0.14
Remdesivir	17(18.68)	9(8.65)	0.039
Tocilizumab	34(37.36)	41(39.42)	0.76
